# Metabolomics-Based Approach for Coffee Beverage Improvement in the Context of Processing, Brewing Methods, and Quality Attributes

**DOI:** 10.3390/foods11060864

**Published:** 2022-03-18

**Authors:** Mohamed A. Farag, Ahmed Zayed, Ibrahim E. Sallam, Amr Abdelwareth, Ludger A. Wessjohann

**Affiliations:** 1 Pharmacognosy Department, College of Pharmacy, Cairo University, Kasr El Aini St., Cairo 11562, Egypt; 2 Pharmacognosy Department, College of Pharmacy, Tanta University, Elguish Street (Medical Campus), Tanta 31527, Egypt; ahmed.zayed1@pharm.tanta.edu.eg; 3 Institute of Bioprocess Engineering, Technical University of Kaiserslautern, Gottlieb-Daimler-Str. 49, 67663 Kaiserslautern, Germany; 4 Pharmacognosy Department, College of Pharmacy, October University for Modern Sciences and Arts (MSA), 6th of October City 12566, Egypt; iezz@msa.eun.eg; 5 Department of Chemistry, School of Sciences & Engineering, The American University in Cairo, New Cairo 11835, Egypt; amr.abdelwareth@aucegypt.edu; 6 Leibniz Institute of Plant Biochemistry, Department of Bioorganic Chemistry, Weinberg 3, 06120 Halle, Germany

**Keywords:** adulteration, brewing, coffee, *Coffea arabica*, *Coffea canephora*/*robusta*, metabolomics, quality control

## Abstract

Coffee is a worldwide beverage of increasing consumption, owing to its unique flavor and several health benefits. Metabolites of coffee are numerous and could be classified on various bases, of which some are endogenous to coffee seeds, i.e., alkaloids, diterpenes, sugars, and amino acids, while others are generated during coffee processing, for example during roasting and brewing, such as furans, pyrazines, and melanoidins. As a beverage, it provides various distinct flavors, i.e., sourness, bitterness, and an astringent taste attributed to the presence of carboxylic acids, alkaloids, and chlorogenic acids. To resolve such a complex chemical makeup and to relate chemical composition to coffee effects, large-scale metabolomics technologies are being increasingly reported in the literature for proof of coffee quality and efficacy. This review summarizes the applications of various mass spectrometry (MS)- and nuclear magnetic resonance (NMR)-based metabolomics technologies in determining the impact of coffee breeding, origin, roasting, and brewing on coffee chemical composition, and considers this in relation to quality control (QC) determination, for example, by classifying defected and non-defected seeds or detecting the adulteration of raw materials. Resolving the coffee metabolome can aid future attempts to yield coffee seeds of desirable traits and best flavor types.

## 1. Introduction

Coffee is a major commodity traded worldwide that contributes to the economy of several countries [1]. In some regions, it is recognized as the second most valuable naturally traded product after oil [2], with an estimate of more than 1.6 billion cups of coffee consumed on a daily basis [3]. Coffee production reached 10,314,000 tons in 2020, with coffee consumption reaching 9,997,000 tons in 2020/2021. This production is contributed by ca. 60 tropical and subtropical countries, as reported by the International Coffee Organization [4,5]. The global coffee industry yield is expressed economically to around USD 200 billion annually [6]. Additionally, recent statistics in the Middle East region show that Saudi Arabia and Egypt account for 75,000 and 77,000 tons of coffee consumption, respectively, which accounts for 1.5% of global coffee consumption [7]. 

Coffee habitual consumption is attributed mostly for its central nervous system (CNS) stimulant effect, specifically for its rich caffeine content, in addition to its characteristic aroma and taste. There are more than 120 species of *Coffea*, and coffee is brewed mainly from the seeds of *Coffea arabica* L. and *C. canephora* L. var. *robusta* or *C. robusta*. Therefore, these species are the most important commercial sources in coffee production [8]. Arabica coffee is favored by most consumers because of its richer aroma and flavor compared to robusta [9]. *C. arabica* accounts for 60% of global coffee production, while *C. robusta* accounts for the remaining 40% [7]. Further analysis of the global market revealed that Latin America produces about 60% and 80% of the world’s coffee supply and arabica coffee, respectively [10]. Particularly, Brazilian coffee is historically a premium coffee that accounts for one third (33.3%) of the worldwide production, and about 24.4% of total coffee exported worldwide [11,12]. 

Further evaluation of coffee import worldwide revealed that coffee is typically imported in the form of green coffee seeds [13], reflecting customers’ special patterns of consumption based on domestic roasting and different blends favored in each region, e.g., cardamom addition in the Middle East, adding further complexity to the coffee metabolome [14,15]. Synergism among several metabolites has been reported for natural products bioactivity, including the antioxidant activity as in the case of coffee constituents [16,17]. Variations within the different species, complexity of the coffee’s chemistry, and low levels of most secondary metabolites through being active or contributing to coffee organoleptic characters warrant for the development of sensitive analytical techniques to monitor all these differences [18]. In addition, one of the main concerns in coffee production lies in the adulteration of roasted coffee to gain economic benefit either by blending low-quality coffee seeds or adding other ingredients, i.e., brown sugar, coffee husks, maize, soybean, etc. [19], or mixing robusta of low quality to premium coffee arabica. Toci et al. have recently reviewed the adulteration practices that necessitate an authenticity quality control protocol to determine coffee origin [20]. For all the aforementioned reasons, in-depth phytochemical analysis of natural metabolites with advanced analytical and evaluation techniques of metabolomics is warranted in coffee for proof of quality and efficacy.

A typical platform for plant extract profiling includes chromatographic techniques such as ultra-high-performance liquid chromatography coupled to mass spectrometry (UPLC/MS), which presents an excellent combination of selectivity and sensitivity allowing for the separation of a large number of components with different modes of ionization, i.e., negative versus positive ESI to increase the identified metabolite scores or coverage [21]. UPLC/MS is especially suited for the profiling of medium polar and large molecular weight bioactives, e.g., chlorogenic acids in coffee, whereas gas chromatography–mass spectrometry is more suitable for aroma profiling in coffee targeting low molecular weight and other volatile compounds or their derivatives, e.g., furans and pyrazines [22].

Recently, LC/MS-based metabolomics techniques have delivered an extraordinary combination of selectivity and sensitivity, which can be employed as an effective platform for metabolite profiling. Different MS techniques provide different ionization modes to gain a wide range of identified metabolites [19]. Moreover, UPLC/MS or fluorescence detection (HPLC/FLD) and UV (HPLC/UV) are well suited for secondary metabolite fingerprinting as a powerful analytical technique for natural plant characterization and classification [21]. 

Compared to chromatographic techniques most commonly employed in metabolomics setups, the direct spectroscopic measurement employed in fingerprinting approaches provides a more robust approach with less run time, including ultraviolet (UV), infrared (IR), and nuclear magnetic resonance (NMR), but it is less able to identify a large pool of compounds [23,24,25]. The UV-VIS technique has been previously applied for coffee fingerprinting, where UV spectral bands provided semi qualitative and quantitative analytical information about selected bioactive components, i.e., phenolic acids, methylxanthines, chlorogenic acids, and pentacyclic alcohols. Moreover, as a cheaper, simpler, and non-destructive technique, UV fingerprinting can be considered an alternative tool to UPLC/MS for coffee sample quality control analyses [26,27]. Similarly, infrared Fourier transform (FTIR) spectroscopy is recognized as a direct spectroscopic technique for discrimination between defective and non-defective roasted coffee seeds [23]. It is also noteworthy to find that NMR has been increasingly employed in food omics studies in the last few years [25,28]. Particularly, ^1^H-NMR has been proven as a potential candidate for the authentication of the commercial Brazilian arabica blends composed of roasted coffee [29]. The common factor in all previous techniques lies in the generation of huge datasets which warrant for the application of statistical modelling tools, including principal component analysis (PCA) and orthogonal projections to latent structure discriminant analysis (OPLS-DA). Conventional analytical methods are challenged with several variables such as huge data sets, geographical location, harvesting time, and chemotypes. These models provide an indicator for method reliability and insights into separations between the investigated sample groups as typical in nutraceutical analyses [30].

Since coffee seeds undergo various post-harvesting steps, volatile and non-volatile metabolites are affected, and are consequently adapted to (regional) consumer preferences. In this review, we shed light on the application of different metabolomics approaches based on different analytical platforms, and specifically those coupled with chemometric data processing. The review aims to unravel methods for identifying metabolic profiles and highlight markers responsible for the unique flavor, aroma, and taste of each coffee type in relation to coffee processing and brewing methods. We present the advantages and limitations of each platform in the context of different post-harvesting methods of coffee, i.e., processing, roasting, and brewing methods, and discuss metabolic profiling-based quality attributes compared to conventional analysis where possible.

## 2. Metabolomics Applications in Coffee Breeding and Origin Determination

A metabolomics-based approach was applied in the classification of coffee seeds derived from different biological origins, i.e., arabica and robusta coffees [22], and conventionally vs. organically grown coffees [31]. Metabolomics has been employed to assess the main chemical differences in these types of coffee, and thus, assign metabolites to the best coffee characteristics. Approaches shall be explained in detail in the next subsections based on the different analytical techniques used.

### 2.1. Gas Chromatography–Mass Spectrophotometry (GC/MS)

GC/MS was employed by Anagbogu et al. [32] to study the different varieties of *C. robusta* in order to determine which genotype should be used in coffee breeding programs to improve its quality. GC–MS analysis revealed 340 metabolites, among which 66 showed differences between genotypes, mainly attributed to sugar derivatives, while the others were organic acids, amino acids and nitrogenous compounds. The study also assessed sucrose to caffeine ratio among genotypes as being indicative of a low cup quality. The germplasm of the ‘Niaouli’ group with a high sucrose/caffeine ratio was recommended for further breeding. 

In another study, a similar metabolomics approach was employed to differentiate between the volatile metabolites of arabica and robusta coffee seeds from different geographical origins in the Philippines in two forms: standard and civet (an animal) eaten forms. PCA was used to model the dataset accounting for 31% of the sample variance, and this identified that arabica samples passed through the civets intestine were enriched in acetic acid, furfural, 2-acetylfuran, 5-methylfurfural, furfuryl alcohol, 3-methylcyclopentane-1,2-dione, maltol, and 2-formylpyrrole, while robusta showed higher levels of 3-ethyl-2,5-dimethylpyrazine, 2-ethyl-3,5-dimethylpyrazine, guaiacol, phenol, 4-ethylguaiacol, and 3-acetylanisole. Likewise, similar metabolites were identified in arabica and robusta coffee seeds when compared to the metabolic profiles of other coffee grown from regions outside the Philippines, mostly attributed to Maillard products, i.e., pyrazines and furans [33].

Similarly, a non-targeted GC/MS metabolite profiling was performed on coffee seeds of *C. arabica* and *C. robusta* from different geographical origins within Indonesia. PCA analysis explained 52.9% of the samples’ variance. This is higher than that found in previous studies and revealed that arabica samples contained higher levels of malic acid, whereas robusta showed higher levels of caffeine, while 16-methyl cafestol was suggested as a discriminant metabolite exclusively present in *C. robusta*. 

Moreover, the assessment of geographical origin effects on metabolic profiles was investigated for Indonesian coffees derived from different species and geographical origins using a non-targeted approach. It was found that Sulawesi, Papua, Flores and Sumatra coffee samples showed higher levels of glycerol, glucuno-1,5-lactone, gluconic acid and sorbitol. Galactinol and galactitol were indicated as metabolite markers to discriminate Sumatra, Bali and East Java from the Eastern parts of Indonesia, with the latter showing higher galactitol levels [34]. Galactitol is a sugar alcohol and its accumulation in the human body has yet to be investigated. 

In another study, GC-Q/MS coupled to multivariate data analysis was employed to determine metabolite markers to differentiate *C. arabica* samples grown in Brazilian coffee producing municipalities, namely, Lavras, Santo Antônio do Amparo (SAA), and São Sebastião da Grama (SSG). PCA explained 51.6% of the samples’ variance, with SAA samples to show higher levels of organic acids, i.e., oxalic acid, malic acid, and sugars, i.e., glucose, fructose, sorbitol, and galactinol. In contrast, quinic acid, caffeine, and 5-caffeoylquinic acid (5-CQA) showed higher levels in Lavras samples. Citric and glutamic acids had higher levels in SSG samples [35]. Independently, blood pressure lowering effects have been implied for glutamic acid-rich foods [36].

### 2.2. LC/MS

As previously mentioned, coffee taste and flavor are important criteria in determining quality. They are strongly influenced by coffee genotype and geographic origin. In a study by Choi et al. [37], an integrated metabolomics approach was performed on coffee samples of different origins representing three continents (Asia, South America, and Africa) using LC/MS along with total proteins, total carbohydrates, and total sugars quantification. Multivariate data analysis showed that monosaccharides, proteins, volatile components and to a lesser extent disaccharide were the most important discriminant factors of the coffee samples and accounted for ca. 65% of metabolite variations. Though, sugars as primary metabolites are not strong markers as they are related to several other factors, e.g., weather. Likewise, Gamboa-Becerra et al. [38] used a metabolome-wide association study (MWAS) employing ultra-performance liquid chromatography–ion trap–mass spectrometry (UPLC–IT–MS) for the analysis of 40 varieties of *C. robusta,* comprising a total of 120 coffee plants. About 91 metabolites were identified as major contributors in determining coffee flavor and taste, which were classified in 11 chemical groups including alkaloids, carbohydrates, carotenoids, chlorogenic acids, fatty acids, flavonoids, lipids, organic acids and terpenoids. Interestingly, quercetin-4-glucoside was found to exert a positive correlation with acidity and sourness and a negative correlation with flavor and aroma, while 52 lipids were found to have a positive correlation with coffee flavor, color, and foam. Foam is a desired feature in most coffee brews and attempts to improve constituents contributing to that factor ought to be further investigated.

### 2.3. Direct Spectroscopic Techniques, i.e., NMR

NMR fingerprinting represents a valuable tool in the classification of the different coffee samples based on their origin. Quantitative ^1^H-NMR (qNMR) methodology was proven efficient for discriminating between numerous arabica samples collected from different regions of Brazil, including São Paulo (North), Minas Gerais (South), Paraná (Tomazina-North), Bahia (Vitória da Conquista-South), and Paraná (Ribeirão Claro-North). Catechol, trigonelline, caffeine, and *N*-methylpyridine are important markers in differentiation [39].

**Figure 1:**
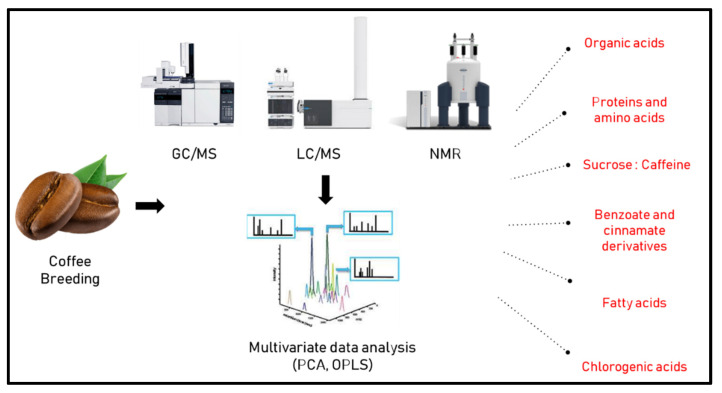
Metabolic class determinants affected by coffee breeding can be determined by different metabolomics approaches.

In another study by Arana et al. [40] ^1^H-NMR fingerprinting was used to discriminate between 192 samples from different origins, i.e., Asia, Africa and America, belonging to arabica and robusta species in comparison to Colombian counterparts with more than 300 spectra collected. Partial least square discriminant analysis (PLS-DA) was then employed to classify samples, with discrimination among samples attributed largely to fatty acids, acetate and caffeine levels. A similar approach was used by Choi et al. [41] using direct NMR spectroscopy for the classification of coffee samples from different origins revealing that chlorogenic acid, caffeine, citrate, and sucrose were the most discriminant metabolites showing higher levels in Colombian samples in comparison to other samples from around the world. These metabolites in turn were suggested by the authors as factors for determining the impact of plant regions on metabolite concentrations. Figure 1 lists various factors and metabolite classes which are likely to affect the choice of coffee species for breeding, as revealed using different metabolomics approaches. We would like to point out that contents of primary metabolites such as sugars, but also some secondary metabolites, strongly depend on weather which may differ between regions and year to year, and thus for reasonable comparisons of different regions several seasons should be sampled, ideally.

## 3. Metabolomics Applications in Coffee Roasting

Upon roasting, the chemical composition of raw coffee seeds undergoes massive transformation processes such as esterification, thermal isomerization, acyl migration, dehydration, and lactonization (epimerization) [42]. Furthermore, in the roasting process part of the carbohydrates get degraded to mono and oligosaccharides (low molecular weight compounds) and interact with amino acids yielding Maillard reaction products such as melanoidins and pyrazines to affect coffee color, flavor, and aroma significantly [14,43].

Further processing of ground, roasted coffee by water-extraction and spray-drying or freeze-drying produces instant coffee products. Such treatment is often associated with a reduction in certain (volatile) compounds such as 2-furylmethanol and low molecular weight organic acids, i.e., acetic, tartaric, malic, formic, and 2-oxo-butyric acid. In contrast, sugars (i.e., sucrose, ribose, and myo-inositol), in addition to acids (i.e., fumaric, propionic, glycolic, and malonic acids) were found to be more enriched in instant coffee compared to ground, roasted coffee products [44]. These variations in acid composition were in part attributed to the processing method of coffee.

### 3.1. GC/MS

In one study, the metabolite profiles of various robusta coffee seeds from Vietnam and Indonesia with different roasting degrees were analyzed using GC/MS and followed by multivariate data analysis. PCA modelling of the acquired dataset explained 75% of the sample variations. Metabolites such as 2,5-dimethylpyrazine, 2,6-dimethylpyrazine, 2-ethylpyrazine, 2-ethyl-6-methylyrazine, 2-ethyl-5-methylpyrazine, dihydro-2-methyl-3-(2*H*)-furanone, and 5-methyl-2-furancarboxaldehyde decreased with roasting degree. In contrast, phenol derivatives (e.g., 2-methoxyphenol), phenol, and 4-ethyl-2-methoxypheno, as well as 2-hydroxy-3-methyl-2-cyclopenten-1-one, and 2,2′-oxybis(methylene)bisfuran exhibited increased levels upon roasting [45].

### 3.2. LC/MS

Chlorogenic acids were annotated in various green coffee specimens based on LC-MS^4^ patterns of fragmentation, including derivatives of dimethoxycinnamoylquinic acids, caffeoyl-dimethoxycinnamoylquinic acids, and diferuloylquinic acids [46], in addition to hydroxycinnamoyl amides [47]. Additionally, roasting changes the coffee chemical profile and increases its complexity, due to the formation of quinides, chlorogenic derivatives, and shikimates. The application of LC in roasting detection in either arabica and robusta roasted coffee via UHPLC-ESI-qTOF-MS/MS mostly targets the visualization of changes incurred by phenolic acids, which are not detected using GC/MS. These include caffeoyl, feruloyl, and diferuloyl quinides described as chlorogenic lactones (CGLs). They are formed by roasting through an esterification reaction on all hydroxyl groups, including C1, followed by dehydration and the formation of lactones which contribute to coffee flavor [48]. These are associated with health effects. Compared to chlorogenic acids, CGLs exert lower antioxidant effects as determined using in vitro DPPH and FRAP assays, although this has yet to be confirmed using in vivo models [15].

Likewise, during coffee roasting, shikimate and lactone derivatives of feruloylquinic acids are also formed. Jaiswal et al. investigated commercial roasted robusta coffee samples using LC/MS and could discriminate between them based on their CGLs and hydroxycinnamoyl shikimates composition [49]. Chemical analysis revealed the presence of various hydroxycinnamoyl shikimates such as *p*-coumaroylshikimic acids, feruloylshikimic acids and caffeoylshikimic acids as characteristic metabolites for roasted coffee seeds.

Pérez-Míguez et al. reported on a non-targeted metabolomics approach based on LC/MS for the identification of metabolites in arabica coffee seeds roasted at different degrees. Chemical compounds that could distinguish between the different degrees of roasting in coffee included caffeoylquinic acid, chlorogenic acid lactones, and *N*-caffeoyl tryptophan which showed a marked increase. The opposite trend was seen for caffeoyl feruloyl quinic acid, mozambioside, coumaroyl quinic acid, and dicaffeoylquinic acid [50]. 

Metabolomic analysis has revealed that caffeoylquinic acid isomers are the major chlorogenic acids; mainly 5-CQA is naturally present in coffee and accounts for its antioxidant and slimming effect. It shows a 33% decline upon roasting, concurrent with an increase in 3-and 4-caffeoyl isomers, though with 5-CQA still being the major isomer. HPLC analysis along with antioxidant activity was performed to evaluate the effect of roasting on the antioxidant activity of caffeoylquinic acid, revealing that antioxidant activity of medium-roasted samples was doubled compared to green coffee, likely attributed to melanoidins generated during roasting and to overcome the loss in chlorogenic acid’s antioxidant action. During roasting, a portion of chlorogenic acid undergoes hydrolysis to form lactones and Maillard reaction products which contribute to the seed antioxidant activity [51]. Melanoidins are a major product of roasting and warrant deeper investigations as scarce information exists on structural changes and technical effects of roasting. Detection and identification of melanoidin levels using LC/MS should aid the optimization of the roasting process of coffee, and likewise for other thermal-processed products, e.g., nuts etc.

### 3.3. Direct Spectroscopic Techniques, i.e., NMR, UV, and IR

To provide a fingerprint of the extract, direct spectroscopic devices are typically used, including UV, IR, and NMR, though with the latter being most powerful in structural elucidation, especially if two-dimensional spectra are used which allow deep amalyses even from crude extracts [23,29,52]. The application of IR spectral analysis in quality control and the metabolite fingerprinting of food products such as coffee seeds has been widely used in determining certain attributes. These methods meet the required criteria for food analysis as they are accurate, non-destructive, rapid, reliable and relatively inexpensive [53]. Previous fingerprinting approaches in coffee have included the discrimination between defective and non-defective roasted arabica coffee seeds employing diffuse reflectance infrared Fourier transform spectroscopy (DRIFTS), where spectral regions 1700–1500 and 970–600 cm^−1^ were reported to show higher absorbance intensity in non-defective compared to defective coffee roasted seeds [23]. Moreover, another application of metabolomic fingerprinting in coffee analysis included phenolic compounds and alkaloid analysis in Indonesian arabica and robusta coffee extracts based on FTIR. The study assigned bands at 1280 and 1605 cm^−1^ for chlorogenic acids and 3123, 3011, 1703 and 1653 cm^−1^ for caffeine [54].

**Figure 2:**
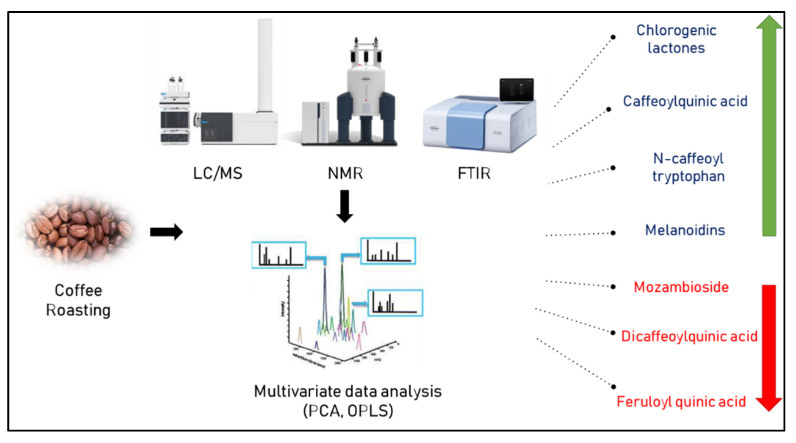
Metabolic markers indicative of roasting degree of coffee as determined by different metabolomic approaches. Green and red arrows indicate increase or decrease upon roasting, respectively.

Furthermore, the authentication of 31 commercial Brazilian arabica blends composed of roasted coffee seeds was successfully performed by ^1^H-NMR based on the signals, particularly between *δ*_H_ 5.1 and 9.5 ppm, specific to carbohydrates of some cereals used for coffee adulteration [29]. Figure 2 lists the various metabolite markers for assessing the degree of coffee roasting as determined by various metabolomic approaches. 

## 4. Metabolomics Applications in Coffee Brewing

Coffee brewing is a historical coffee preparation method employed using various extraction methods, including drip, French press, boiled brew, pressurized extraction (espresso), and others [54,55]. These forms can be performed by different devices ranging from traditional brewing pots to automatized coffee makers resulting in various coffee beverages according to several factors, mainly related to consumers’ preferences and acceptability for each region or country [56,57,58]. Cold brewing has gained consumers’ preference recently, whereby coffee is extracted at 20 to 25 °C or colder and steeped for a much longer time ranging from 8 to 24 h, compared to the classical hot brewing methods performed on a minute scale [59]. Beside coffee origin and post-harvest processing, coffee brewing is recognized as a critical step to affect coffee sensory attributes, including flavor and taste [54,60,61]. For instance, Nariño cold brew shows unique sweetness and fruity and floral flavors, in addition to medium bitterness and acidity, with a creamy consistency [62]. Consequently, several studies have attempted to investigate the sensory profiles associated with different brewing methods [63,64], which are yet to be correlated with chemical profiles to be conclusive.

Both volatile and non-volatile metabolites are typical markers contributing to common coffee characteristics, i.e., aroma perception, astringency, and bitter taste [56,65]. This asks for the employment of more than one analytical technique to holistically assess coffee flavor makeup. Furthermore, the preparation of different blends unique to world regions can also contribute to coffee sensory properties during the brewing process and add to its chemical complexity. For instance, blends containing some herbal spices such as cardamom seeds and clove buds are frequently consumed in the Middle East, masking the smoky odor of roasting products, including 5-(hydroxymethyl) furfural (5-HMF) and pyrazines [14]. The brewing method also affects non-volatile bioactives levels in coffee, including caffeine and phenolic acids to account for claimed health benefits of the seeds, i.e., antioxidant and anti-tumor properties [55,66,67,68,69]. Examples include hot extraction methods found to affect the polyphenol and caffeine contents, while further analysis of cold brewing methods, i.e., dripping and steeping, resulted in changes of chlorogenic acid and trigonelline levels [62].

Additionally, extraction conditions in cold brews affect Maillard reaction products such as acrylamide and furans. Hence, the optimization of acrylamide and furan levels is a major goal in coffee brewing to ensure healthier coffee beverages with less hazards. Han et al., showed that coffee brewed for 3 h contained the lowest acrylamide levels, while steeping and dripping featured the lowest furan levels after 24 h and 12 h, respectively [64]. The effect of coffee brewing on microelement composition appears conflicting though, and likely is not significantly influenced by the brewing method, warranting further studies [70]. Janda et al. have reported recently that coffee beverages prepared by simple infusion and AeroPress were rich in magnesium (116.3 mg/L), manganese (0.6 mg/L), chromium (0.03 mg/L), cobalt (0.01 mg/L), and potassium (1540.7 mg/L), while the drip brew contained a valuable silicon content (3.4 mg/L) [71].

**Figure 3:**
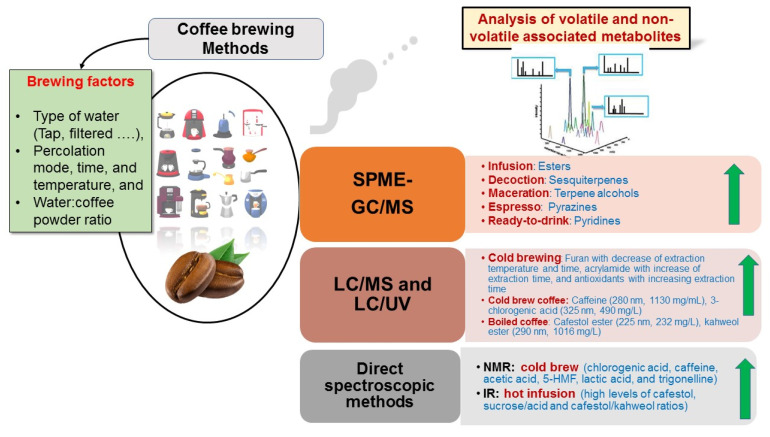
List of the different brewing factors contributing to volatile and non-volatile metabolic profiles of coffee brews. The metabolites markedly increased with specific brewing methods are mentioned based on the detection method used.

Different variables have been determined in previous coffee brewing studies to ensure the best products, including device used such as coffee maker or traditional coffee pot, temperatures, times of percolation, water/coffee ratio, and water type [54,72]. Some studies have targeted few metabolites, such as caffeine, furan, and 5-HMF [73], while metabolomics-based approaches using different platforms, i.e., GC/MS, LC/MS, and NMR could provide better readouts of brewing impact on coffee. GC/MS targets low molecular weight aroma compounds, e.g., terpenes and polar primary metabolites post-derivatization, i.e., fatty acids, sugars, and amino acids. LC/MS and NMR were more effective to detect and identify potential secondary bioactive compounds, including diterpenoids, chlorogenic acid derivatives, and alkaloids [22]. In the context of coffee brewing, various factors and their different platforms are illustrated in Figure 3 and shall be covered in the following subsections highlighting the effects of different brewing methods on coffee metabolomes using different analytical techniques. In addition, the different metabolites that markedly increased with the main brewing method are summarized in Figure 3, along with the platform which could detect and quantify them.

### 4.1. GC/MS

Mostly, headspace solid-phase microextraction (HS-SPME) coupled with GC/MS was employed for investigating volatile constituents or volatile organic compounds (VOCs) associated with coffee brewing. In addition, gas chromatography/olfactometry (GC/O, CharmAnalysis) is sometimes used [74,75,76]. HS-SPME coupled to GC/MS was employed to assess the different brewing methods, including hot infusion, maceration, and decoction, typically reported in the Middle East region and analyzed using chemometric tools to identify markers for each brewing method. HS-SPME improved volatile detection with a total of 102 VOCs. Among VOCs, esters of mainly octyl acetate and terpinyl acetate, sesquiterpenes (e.g., α-curcumene, bergamotene, and *β*-caryophyllene), and terpene alcohols (e.g., terpineol, linalool and octanol) were found to predominate coffee infusion, decoction, and maceration, respectively [14]. 

Using a similar volatile collection setup, Yu et al. assessed the type of water, i.e., filtered, tap, mineral, and bottled, used in the brewing process for *C. arabica* of different roasting degrees by an espresso coffee machine versus the cold brew method. SPME-GC/MS results showed that brewing with so-called “filtered” water in an espresso coffee machine increased pyrazines, i.e., 2-methylpyrazine, 2,5-dimethylpyrazine, and phenols, (i.e., 2-methoxy-4-vinylphenol levels) compared with tap and bottled water. Compared to hot espresso-brewed coffee, cold brew increased the contents of 2-methylpyrazine, 1-methylpyrrole, and 2-acetylfuran, possessing a sweet, nutty, and fruity odor [77]. However, health perspectives have not been investigated based on such chemical makeup.

Moreover, chemometric tools have been applied to comprehensively assess the aroma profile of capsule-brewed espresso coffees derived from diverse Italian brands. Partial least squares discriminant analysis (PLS-DA) revealed that pyrazines (i.e., 2-methylpyrazine, 2,5-dimethylpyrazine, 2,6-dimethylpyrazine, and 2,6-diethyl-pyrazine) are significant coffee markers imparting a characteristic and intense aroma [78]. Heo et al. monitored VOCs derived from different volatile compounds in cold brew coffee samples, including cold brew from a coffee shop, ready-to-drink coffee, and brewed coffee from a coffee maker by HS-SPME/GC–MS. The results demonstrated a total of 36 volatile compounds with higher levels in coffee shop vs. ready-to-drink coffee samples, especially with pyridines imparting bitter, burnt, roasted, and astringent characteristics, such as *N*-acetyl-4-(*H*)-pyridine. Pyridine and its derivatives are likely to be products of trigonelline degradation, especially for seeds roasted strongly at high temperatures [79,80]. In addition, simple phenolics, i.e., guaiacol and its congers, were among the potential markers that characterized coffee shop samples, whereas coffee from a coffee maker showed higher amounts of maltol, which is responsible for the caramel-like odor and chocolate flavor in coffee and other confectionaries [80]. 

Moreover, GC/O of freshly brewed drip coffee (Ethiopian arabica coffee, roast degree: L value; 23, fiber type: divinylbenzene/carboxen/polydimethylsiloxane adsorbant) showed different VOCs, possessing a nutty–roast odor and a raspberry ketone sweet–fruity odor, represented by 1-(3,4-dihydro-2*H*-pyrrol-2-yl)-ethanone and 4-(4′-hydroxyphenyl)-2-butanone, respectively [74]. PCA was applied for the dissipation of VOC profiles to discriminate between various arabica coffee extracts collected from three production countries, Ethiopia, Tanzania, and Guatemala, with different roasting degrees. The results showed that the Ethiopian coffee extract was characteristic by its high amount of 4-(4′-hydroxyphenyl)-2-butanone [81].

### 4.2. LC/MS and LC/UV

LC/MS has been applied for studying the non-volatile metabolites associated with coffee brewing, including polyphenolics, alkaloids, and diterpenes. Compared to roasting effects, the effects of brewing on phytochemical composition, particularly chlorogenic acid and hydroxycinnamoyl amide derivatives, has not been extensively reported [46,47]. LC/MS showed potential in detecting health-related metabolites, either undesirable as acrylamide and furans, or desirable as the antioxidant phenolics, providing a good readout of coffee safety and efficacy of coffee prepared by cold methods [64,82]. The International Agency for Research on Cancer (IARC) classify acrylamide and furan among probable carcinogens (Group 2A) and possible human carcinogens (Group 2B), respectively [83,84]. Therefore, levels of acrylamide should be accurately determined by sensitive analytical techniques, including LC–MS/MS [82] and they are typically in the range of 5.9–38.8 ng/mL in ready-to-drink (brewed) coffees [83]. Moreover, furan levels in brewed coffee should be near or below 120 μg/L [84]. The effect of cold brewing on health hazard-related metabolites showed that acrylamide levels increased with increasing extraction time (5.5 ± 0.4 ng/mL for steeping after 24 h at 20 °C and 5.2 ± 0.4 ng/mL for dripping after 12 h at 20 °C), whereas a decrease in furan levels was observed with increasing extraction temperature and time (10.1 ± 0.2 ng/mL for steeping after 24 h at 20 °C and 12.3 ± 0.3 ng/mL for dripping after 12 h at 20 °C). Total phenolic content increased with increasing extraction time and independently from brewing method, especially with samples prepared for 24 h steeping at 20 °C, 18.9 ± 0.3 µmol GAE/mL [64,82].

In addition, HPLC coupled to a diode array detector (HPLC-DAD) at 280 nm and 325 nm was used to target key coffee constituents, i.e., caffeine and 3-chlorogenic acid (3-CGA), respectively, in the context of the different extraction parameters, including time, roasting temperature, and grind size, in cold and hot brews [85]. After 400 min of brewing time, results revealed that 3-CGA was more abundant in cold brew coffee made with medium roast coffees, detected at a concentration of 490 ± 30 mg/L. Moreover, caffeine showed higher levels in cold brew of coarse grind samples (1130 ± 50 mg/L) than in their hot counterparts [86]. HPLC–DAD combined with spectral deconvolution showed potential for the quantitation of different coffee brews’ diterpenes, i.e., cafestol and kahweol as esters of linoleate, oleate, palmitate and stearate [87,88], adjusted at 225 and 290 nm for cafestol esters and kahweol esters, respectively. Results demonstrated that boiled coffee showed highest diterpene ester levels (232 mg/L and 1016 mg/L for total cafestol and kahweol esters, respectively), whereas instant brews showed lowest levels at 1.3 mg/L and 2.0 mg/L for total cafestol esters and kahweol esters in instant espresso, respectively [87]. Previous reports showed that cafestol and kahweol are typically found in the range of 182–1308 and 0–1265 mg/100 g, respectively [89]. Diverse health-promoting benefits of coffee, including anti-inflammatory, immunomodulatory, anti-tumor, anti-diabetic, hepato-, cardio-, and neuroprotective effects are attributed to such diterpene content [90]. 

### 4.3. Direct Spectroscopy Techniques, i.e., IR and NMR

Compared to hyphenated chromatographic techniques, i.e., GC/MS and LC/MS, NMR is not commonly applied in coffee brewing studies. However, NMR allowed the successful differentiation between diverse brewing methods, including cold and hot brew, aided by PCA modelling of the full NMR spectra [91]. The result of this study revealed that the levels of chlorogenic acid, caffeine, acetic acid, 5-HMF, lactic acid, and trigonelline were increased with of ultrasonication assisted extraction by 71%, 26%, 21%, 16%, 81%, and 19%, respectively, after a one-hour cold brew extraction compared to an extraction without agitation.

IR was also applied in a few studies on coffee brewing in order to aid the sensory quality evaluation of Brazilian arabica coffee brews [92]. The various coffee brews were prepared by hot infusion and the results showed that green coffee brews with high quality scores were associated with low levels of caffeine and protein chlorogenic acids, in addition to high levels of cafestol, sucrose/acid and cafestol/kahweol ratios [92]. 

## 5. Coffee Authentication and Adulteration Detection

Metabolomics approaches are increasingly reported for herbal drug authentication and quality control [93,94]. According to the reports of the International Coffee Organization (ICO, London, UK), coffee adulteration either intentionally or accidentally is a serious issue threatening the coffee market [95], mainly through the replacement of coffee powder with other, cheaper products [31]. More than adulteration detection, the development of methods to detect and identify coffee substitutes is of equal value, as is the discrimination between natural coffee and substitutes, including cereal grains (e.g., corn, barley, soy, oat, and rice) and legumes (e.g., lupin seeds and nuts) or chicory root [96,97]. Practically, authentication approaches are always based on either targeting specific markers of adulterants, or through the non-targeted fingerprinting approach [98]. Different metabolomics-based platforms were applied, including direct NMR spectroscopy, infrared/Raman spectroscopy, and UV-Vis versus hyphenated chromatographic technique coupled to mass spectrometry (i.e., GC/MS and LC/MS) [99], as highlighted in the next subsections. 

### 5.1. GC/MS

Static headspace GC–MS (SHS–GC/MS) combined with chemometric tools was applied for the authentication of elephant dung coffee (Black Ivory Coffee) via its volatile profile [100]. Elephant dung coffee is a unique non-bitter Thai coffee derived from *C. arabica* collected from feces after consumption by Asian elephants to improve its organoleptic properties via fermentation in the animal gut [101]. Among 78 identified VOCs, 3-methyl-1-butanol, 2-methyl-1-butanol, 2-furfurylfuran, and 3-penten-2-one have been recognized as potential discriminant markers for elephant dung coffee [100].

Authentication and quality control through the non-volatile constituents can be also carried out by GC/MS, but after derivatization. Considering the premium quality of Arabic versus robusta coffee, the identification of markers for each type is warranted for QC purposes. GC/MS post silylation profiling, targeting nutrients in arabica and robusta, led to the detection of 143 metabolites, with arabica seeds found to be more enriched in fatty and organic acids, i.e., palmitic acid and acetic acid, respectively. In contrast, robusta was more rich in cyclitol sugars, i.e., myo-inositol and sorbitol [22]. However, these markers need to be confirmed by analyzing several other specimens of other origins, considering that macronutrients are highly affected by plant agricultural conditions and ecological backgrounds, as pointed out earlier.

### 5.2. LC/MS and LC/UV 

LC-based quality control of coffee products has been comprehensively reviewed previously, and it is considered a targeted analytical platform using carbohydrates and other phenolic compounds as markers [99]. Moreover, flavoromics-based untargeted multidimensional preparative LC/MS analysis, with the aid of 1D- and 2D-NMR, could reveal different metabolites that positively and negatively affect coffee quality based on cup score. Three compounds were reported to be associated with an increase in coffee quality, including 3-*O*-caffeoyl-4-*O*-3-methylbutanoylquinic acid, 3-*O*-caffeoyl-4-*O*-3-methylbutanoyl-1,5-quinide, and an unknown phenolic derivative containing a 3-methylbutanoyl moiety [102]. In contrast, six metabolites were associated with low cup score, including *ent*-kaurane diterpenes, i.e., 16*α*,17-dihydroxy-*ent*-kauran-19-oic acid, 16*α*,17-dihydroxy-*ent*-kauran-19-diglycoside, 16*α*,17,18-trihydroxy-*ent*-kauran-19-oic acid, and 16*α*-hydroxy-17-*ent*-kauren-19-oic acid reported from green seeds. These findings were observed from the analysis of *C. arabica* cultivated in different countries worldwide [103]. Furthermore, Gao et al. attempted to modify the bitter flavor of coffee brew through the identification of the bitter modulators with the aid of untargeted LC/MS profiling combined with descriptive sensory analysis and modelling using OPLS. 4-CQA, 5-CQA, and 2-*O*-*β*-d-glucopyranosyl-atractyligenin were verified to suppress the bitterness perception [104], in agreement with Blumberg et al.’s findings [105]. 

Moreover, the antioxidant power of coffee seeds has been successfully related to metabolites tentatively identified by UHPLC-ESI-HRMS. PLS-DA showed that caffeoylquinic acids and caffeine are mostly related to the antioxidant effects of unroasted coffee, while dicaffeoyl quinolactone and melanoidins are responsible in roasted coffee [15].

Furthermore, UPLC-HRMS was used for coffee adulteration detection based on the oligosaccharide profiling of common adulterants, i.e., soybeans and rice, in ground coffee as low as 5% [96]. About 17 oligosaccharides were identified and calculated by Glycoworkbench as potential candidates, including Hex5-Hex14 and Hex2Pen4&Hex5AcHex of molecular masses in the range of 0.9–2.0 kDa. 

Untargeted high-performance liquid chromatography coupled to ultraviolet (HPLC–UV) fingerprinting analyzed using PLS-DA led to the detection and estimation of coffee frauds by mixing coffees of different geographical origin. The results showed a detection and quantitation of the adulterant levels down to 15%. Moreover, the validation parameters, including calibration, cross-validation, and prediction errors which were determined to be below 2.9%, 6.5%, and 8.9%, respectively [19], were suggestive of no model overfit. Moreover, HPLC coupled to ultraviolet and fluorescence detection (HPLC–UV–FLD) was employed for the detection of three common adulterants, including chicory, barley, and flours. The proposed methodology could assess coffee authenticity and quantify adulteration levels down to 15% [106].

### 5.3. Direct Spectroscopy, i.e., NMR, UV, and IR

Despite NMR’s potential as a metabolomic tool for marker identification and strong structural elucidation, it is less used compared to GC/MS and LC/MS [107]. qNMR could discriminate between arabica vs robusta, in addition to the prediction of robusta percentile in coffee blends based on the quantification of alkaloids (e.g., caffeine, trigonelline), caffeoylquinic acids (e.g., 3- and 5-CQA), diterpenes (e.g., cafestol, kahweol, and 16-*O*-methylcafestol (16-OMC), and organic acids (E.G., acetic acid). The results showed that diterpenes are potential discriminators; 100% of arabica seeds had low 16-OMC, while robusta had low kahweol content [108]. Furthermore, *β*-ethanolamine has been reported as roasting marker in arabica and robusta, as revealed by NMR aided with multivariate data analyses [22].

Increasing robusta level in coffee mixture has been found to lower its quality compared to pure arabica coffee. Compared to arabica seeds, robusta is more abundant in caffeine and myo-inositol [22] and can rationalize the enrichment of caffeine in instant coffee products being produced mostly from robusta seeds [69]. Interestingly, modelling of the coffee NMR dataset resulted in a stronger mathematical model, evident from variance coverage and prediction power in comparison to GC/MS data, applied in the discrimination between instant coffee from other roasted products [22]. In addition, authentication of commercial Brazilian arabica blends composed of roasted coffee seeds was effectively based on ^1^H-NMR through the identification and quantification of common coffee adulterants, including corn, coffee husks, barley, and soybean. The results revealed that the spectral region *δ*_H_ 5.1 to 9.5 ppm provided potential signals that can be used for differentiation between coffee samples and the adulterants, based mostly on the anomeric proton signals of carbohydrates [29].

Moreover, UV fingerprinting with the aid of multivariate data analysis models demonstrated high absorption values, particularly at 350–450 nm, assigned to melanoidin content in roasted seeds and blended coffee products as often consumed in the Middle East region [15]. Moreover, UV-based metabolomics has been proven as a potential alternative for LC/MS and a non-destructive analytical tool for the identification of the roasting-induced toxins, especially acrylamide. Its detection was confirmed by the spiking method, recording an increased optical density at 273 nm, in agreement with Alfarhani [15,109]. 

Furthermore, previous reports have reported the use of IR for the detection of adulteration in coffee products. For instance, Winkler-Moser et al. authenticated the Brazilian roasted coffee via near infrared (NIR) in the presence of roasted, ground corn based on their tocopherol content in a sensitivity as low as 5% [110]. Moreover, the detection of multiple adulterants, including corn, barley, soybean, rice, and even coffee husks and robusta coffee by NIR in roasted and ground arabica coffee was possible, derived from different geographic origins. The developed method was able to detect adulterants in concentrations ≥10% [111]. In addition, mid-infrared (MIR) Fourier transform spectroscopy (FT-MIR) coupled with chemometrics showed its identifying and quantifying power for arabica coffee adulterants (e.g., 1–30% of corn, barley, soy, oat, rice and coffee husks). The established chemometric models exhibited influential validation parameters of R^2^c: ≥0.99, in addition to a standard error of calibration (SEC) and standard error of prediction (SEP) of 0.39–0.82 and 0.45–0.94, respectively [97]. Another study used attenuated total reflectance–Fourier transform infrared spectroscopy (ATR-FTIR) of the mid region for the detection of four adulterants, i.e., spent coffee grounds, roasted coffee husks, roasted corn, and roasted barley, simultaneously. The validation parameters were improved based on two approaches, including hierarchical models (HM) and data fusion (DF) reaching to 0% misclassified samples in the second level models [112].

## 6. Conclusions

Coffee brewing is the last step of coffee post-harvest processing to affect aroma and flavor. Various techniques are used, based mostly on consumer and cultural preferences. SPME coupled with GC/MS is the most commonly used platform to unravel volatiles behind the unique aroma, which are mainly generated during roasting processing. Other analytical platforms, including NMR and LC/MS proved suitable for profiling non-volatiles in coffee, particularly cinnamoylquinic and feruloylquinic acid derivatives, warranting the employment of comparative metabolomics approaches to assess coffee composition. Metabolomics-based authentications of coffee products are relatively novel techniques depending on non-targeted analytics combined with chemometric tools. Compared to authenticated coffee seeds of different botanical and geographic origins, roasting degrees, production methods, and blends with other aromatic herbs, it allows the detection of governing molecular components and marker compounds. UV-based metabolomics has gained a particular interest as an alternative tool to the powerful LC/MS for potential marker and toxin identification, including the identification of the carcinogenic roasting metabolite acrylamide. Therefore, metabolomics investigation could successfully and comprehensively assess coffee products for quality control purposes, although it has yet to be applied at commercial levels. Compared to hyphenated techniques, direct spectroscopic measurement (especially simple and cheap IR) appears more suitable for local industrial applications monitoring consistency among batches, similar to methods routinely performed in drug analyses aided by chemometric tools.

## Data Availability

Not applicable.
